# Depression detection with machine learning of structural and non‐structural dual languages

**DOI:** 10.1049/htl2.12088

**Published:** 2024-06-10

**Authors:** Filza Rehmani, Qaisar Shaheen, Muhammad Anwar, Muhammad Faheem, Shahzad Sarwar Bhatti

**Affiliations:** ^1^ Department of Computer Science & Information Technology The Islamia University of Bahawalpur Bannu Pakistan; ^2^ Department of Information Sciences, Division of Science and Technology University of Education Lahore Pakistan; ^3^ School of Technology and Innovations University of Vaasa Vaasa Finland; ^4^ Department of Computer Science Emerson University Multan Pakistan

**Keywords:** artificial intelligence, machine learning, healthcare, depression detection, languages

## Abstract

Depression is a serious mental state that negatively impacts thoughts, feelings, and actions. Social media use is rapidly growing, with people expressing themselves in their regional languages. In Pakistan and India, many people use Roman Urdu on social media. This makes Roman Urdu important for predicting depression in these regions. However, previous studies show no significant contribution in predicting depression through Roman Urdu or in combination with structured languages like English. The study aims to create a Roman Urdu dataset to predict depression risk in dual languages [Roman Urdu (non‐structural language) + English (structural language)]. Two datasets were used: Roman Urdu data manually converted from English on Facebook, and English comments from Kaggle. These datasets were merged for the research experiments. Machine learning models, including Support Vector Machine (SVM), Support Vector Machine Radial Basis Function (SVM‐RBF), Random Forest (RF), and Bidirectional Encoder Representations from Transformers (BERT), were tested. Depression risk was classified into not depressed, moderate, and severe. Experimental studies show that the SVM achieved the best result with anaccuracy of 0.84% compared to existing models. The presented study refines thearea of depression to predict the depression in Asian countries.

## Graphical abstract

The risk of depression is classified into three categories not depressed, moderate depression, and severe depression. Out of these three models, SVM achieved the best result with an accuracy of 0.84%. The proposed work refines the area of depression prediction and prove to be an excellent step to predict the depression in the Asian countries.

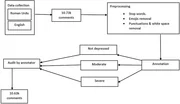

## 1 INTRODUCTION

According to World Health Organization (WHO) [1], depression is a common and execrable mental state. More than 280 million people worldwide suffer from mental illness called “Depression.” An estimated 3.8% of the world's population is affected by this dementia, including 5.7% of people over the age of 60 and 5.0% of adults. Depression is a crucial element of suicide. Patients who suffer from mental illness for a long time have a tendency to commit suicide. All around the world suicide is in fourth place among the causes of death of 15–29 years old. Suicide is a global problem that is prevalent not only in developing countries, but also in developed countries. In 2019, 77% of suicides were committed in developing countries. Depression is the main reason due to which people tend toward suicide or commit suicide. An estimated 75% of people in developing countries who suffer from depression are untreated.

According to Healthline, approximately 60% of people attempt self‐murder (suicide) because of major depression, bipolar disorder, and mood disorders [2]. In this modern era, advancements in technologies have attracted many people, due to which social media has gained an important place in people's lives. The total number of monthly active users on Facebook currently reaches 2.9 million and Twitter has 397 million active users [3]. Individuals express their thoughts, feelings, interests, and routine lives by posting on social media, for the sake of sharing, liking, and commenting on the posts. All these social media platforms, including Facebook, Twitter, Reddit, Instagram, and many others, provide a lot of help to researchers in terms of data collection. These social media platforms are an important source of data for research. Substantial work has been done on depression prediction in English and is still being done. Apart from English, a lot of work is being done in Urdu and other structural languages.

The South Asia region is formed from eight countries. In 2010, Muslims, Hindus, and Sikhs population were declared as the world's largest population, in the South Asia region. Users usually use their regional languages for expressing their thoughts on social media. Muslims, Hindus, and Sikhs who live all over the world, especially in Pakistan and India, use their regional language, “Roman Urdu” to communicate with each other on social media platforms or to share their feelings and interests.

Urdu written in Latin script or Urdu written in the English alphabet is called “Roman Urdu.” It is also called the Latin Urdu script. In Turkey, Atatürk chose the Latin alphabet script for the Turkish language. Inspired by this General Ayyub Khan during his rule in the country seriously proposed to adopt the Latin alphabet script for Urdu and all other languages spoken in Pakistan. In south Asian regions, Roman Urdu is very famous among the people through which they can communicate with each other on digital media platforms such as Twitter, Facebook, Instagram, and SMS.

It has been observed that people in Asian countries often use Roman Urdu for writing text messages, especially in Pakistan and India, on different social networking sites. Christian living in Asian countries, especially in Pakistan and India, use the Roman script to write Urdu. In India, the Bible is published in Roman script, and the songbooks kept in churches are also written in Roman Urdu.

There are no such restrictions on spelling in Roman Urdu, it will not be wrong to say that one word can be written with different spellings. Not only can different people write different spellings, but the same person can write different spellings at different times or at the same time as well [4]. In Roman Urdu a single word contains many variations in it. Spelling variation, for example, Umeed “HOPE,” can also be written as “Umed,” “ummed,” “ummeed” as well [5].

Gilani Research Foundation (GRF) survey conducted by Gallup Pakistan shows that 37% of people claim that they used Roman Urdu (Urdu in English alphabets) to text anyone by using cellphones. 15% users used Urdu (Arabic script) to send text; 17% said that they often send messages in English; 29% claim that they do not even send messages and 2% cannot show any response toward this survey [6]. However, most of the research related to depression prediction has been driven by using English corpus/datasets from different social media platforms. There are many datasets, questionnaires, and surveys available to predict depression in English and many other structural languages. To the best of our knowledge, there is no dataset, and no technique has been used to predict depression in Roman Urdu.

By analysing previous literature, we were unable to find any significant contribution to predict the depression through Roman Urdu (non‐structural language), or Roman Urdu (non‐structural language) along with another (structural languages) like English. It means that predicting depression in non‐structural or low resource languages is perhaps more challenging than in structural languages. The main reason is the lack or unavailability of the dataset. All the datasets that are available in English cannot be used to train the data for non‐structural languages, which limits their functional implications.

Regarding the proposed methodology, to predict depression in Roman Urdu, we have investigated the most commonly used machine learning models: Support Vector Machine (SVM), Support Vector Machine Radial Basis Function (SVM RBF) and Random Forest (RF). We have trained these models using extracted features, and SVM achieves the best result with an accuracy of 80% in predicting depression by using structural and non‐structural languages.

The prime additions of this study are as demonstrated:

- A Roman Urdu benchmark dataset has been manually created. In which there are 3k Roman Urdu comments.
- Second Roman Urdu + English benchmark dataset has been created. In which there are 10.73k comments. To predict depression through dual languages.
- We manually annotated the dataset and classified it into three labels: moderate, not depressed, and severe.
- We have used count vectorization to extract the features so that we can increase the accuracy of the models and reduce the training complexity.

The layout of the paper is structured as follows:

Section 2 comes up with a review of related work. Section 3 introduced the proposed model and flow chart of the proposed method, machine learning techniques and implementation steps for investigating the proposed model. Section 4 discloses the results of investigated models. Section 5 contains the discussion about the resultsand the conclusion is finally described in Section 6.

## 2 LITERATURE REVIEW

The use of social media is increasing rapidly in this time and age. For the sake of communication, people share their feelings, viewpoints, and different aspects of life on different social media sites. Many researchers predict different psychological trails such as anxiety, depression and stress levels through their posts and different social media activities [7]. People share their feelings, ideas, images and videos on social media without knowing the positive and negative impact [8].

Depression is a complicated and serious mental illness among people these days. Till date, the main or exact causes of depression have not been known. Most people who are going through depression commit suicide. The leading cause of suicide is untreated depression. Comparatively, [9] (Chiong, R., 2021) proposed a method to predict depression among the social media users by examining their posts, especially when the posted text does not contain any definitive keywords such as “depression” or “diagnosis.” According to the WHO around 280 million people are suffering from depression [10].

Many techniques of, machine learning, deep learning and NLP, have been used to predict depression related to our research, which are described in detail below.

Natural language processing (NLP) has revealed its benefits in many fields. Apply NLP (lexicon‐based approach) along with machine learning models to identify depression symptoms from Arabic text data [11]. NLP techniques and ML approaches are applied to train the data. Data is collected from Reddit. Combine features achieve an accuracy of 91% with multilayers perceptron (MLP) and 0.93% of F1 score. The best character of a single feature is bigram with SVM, which achieves 80% accuracy and a 0.97% F1 score [12].

Many deep learning methods have been used to identify depression. Wang et al. proposed deep learning methods used with pretrain models such as, BERT, ROBERTa and XLNET to predict the risk of depression. In which the risks of depression are categorized in four different levels 0–3. 0 is denoted with no inclination, 1 with mild depression, 2 with moderate depression and 3 with severe depression [13]. A productive method along with LSTM and RNN was introduced to identify the symptoms of depression. The text data for prediction were collected from questionnaires and views posted by the younger generation on an informational channel. A deep learning approach was used for time sequential features. This approach achieves performance of 98% and 99% [14].

Neural models (CNN) are proposed to predict depression by using Chinese microblogs [15]. KNN, SVM and Fine‐Tree algorithm is analysed to study the individual's tweets to predict depressed and non‐depressed tweets [16]. A BPNN (back propagation neutral network) model [17], was introduced to identify the depression. The risks of depression, depression is divided into four categories, normal, mild, moderate, and severe. For this purpose, the data of 227 patients was obtained. This obtained data set is divided into two parts: one is testing and the other is training. Out of 227 data, 80% were used for training and 20% for testing purposes. The F1 score of normal was 100%, mild was 95.65%, moderate was 90.91%, and severe was 95.24% and the obtained accuracy is 95.65%.

Automatic depression detection (ADD) is the most important point in identifying depression [18]. Because human actions, his voice, and words, indicate his state of mind. Therefore, text, facial expression, speech, text and voice features, facial actions and voice have also been used to predict depression. A temporal pooling method was introduced to automatically detect depression through facial expressions by using videos [19]. Gaussian mixture modelling combined with factor analysis was introduced in [20]. To achieve the goal of predicting depression in speech, 35‐speaker free response speeches were used as dataset. The result of baseline is constantly upgrading. These models achieve the best result with 95% confidence with small datasets.

A multimodal approach was used to detect depression through text and voice automatically in [21]. An interview corpus (DAIC) was used as a text dataset. This interview is conducted by a computer agent for the ease of participants. In a room, a large computer screen was arranged for virtual interviews. A voice quality model was proposed that operates on the voice of participants during the interview. Model for text analysis achieve 0.81% F1 score and 0.70% accuracy. The model for voice quality achieved an accuracy of 0.66% and 0.75% F1 score.

Active appearance modelling (AAM) combined with FACE and pitch extraction were proposed for facial and vocal expressions. SVM is used for FACE and AAM and for vocal prosody logistic regression was used. To achieve this objective, 50 people were interviewed. FACE achieved an accuracy of 88% and AAM achieved 79% [22].

Few works have also been done on multi‐languages to predict depression. This [23] study is divided into two parts. A depression post classification model was introduced for three languages: Korean, Japanese and English, which play an important role in predicting depression in multilingual. After that, a depression lexicon was created for each language. To achieve this goal, data has been obtained from Tweeter.

This study focused on three languages—German, Hungarian and Italian to predict depression. For German language (AViD corpus) is used. Voices were recorded at different values of BDI for the Hungarian database, and recordings from eleven different speakers were used for Italian. A quasi‐language independent system with SVR was used. Overall study experiments achieve an accuracy of 86% [16].

Cross cultural depression prediction has also been given special importance. In which a comparison was made to find out the risk and levels of depression, among the people belonging to different countries, having different languages and cultures. In order to find out which country or which language speaking people have higher rates of depression [24].

According to the first census conducted by the “Pakistan Bureau of Statistic” in 2017 after 1998, only 7.08% of people in Pakistan use Urdu as their mother tongue [25]. “Ethnologue” publishes a list of ‘‘most spoken languages 2022,″ where Urdu is the tenth most spoken language in the world [26].

In order to predict depression, special attention has been given to structural languages such as English, apart from structural language, many non‐structural languages has also worked on. In Asian countries, people communicate with each other in Roman Urdu on social media, which has not been used to predict depression.

## 3 MATERIALS AND METHODS

**FIGURE 1:**
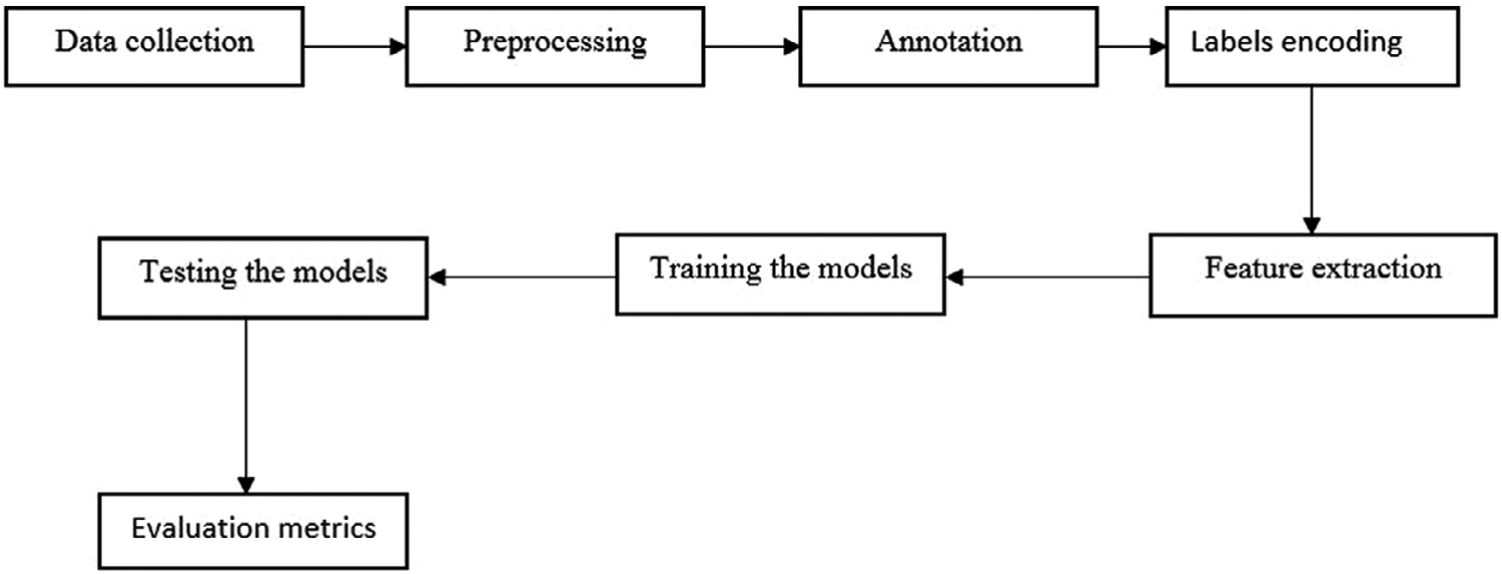
The flow chart of the proposed methodology.

In this study, a methodology, shown in Figure 1, has been introduced, that predicts depression in structural and non‐structural dual languages.

The framework is implemented in eight sessions: data collection, data preprocessing, manual annotation, label encoding, feature extraction, training and testing, classifiers, and matric evaluation.

### 3.1 Data collection

For this research, two datasets have been obtained from two different social media platforms, for Roman Urdu, English data has been obtained from Facebook, which consists of 3000 comments, which we have manually converted into Roman Urdu. Examples of Roman Urdu comments are given below in Table 1.

**TABLE 1:** Example of moderate, not depressed, and severe in Roman Urdu.

Comments	Labels
M bahut thak gaya hon m bs marna chahta hon	Severe
Kuch waqt ho gaya h nekly huey	Not depressed
Essa mehsos Karyn jessy m depression k ibtadae marahil m hon	Moderate

While the second dataset which consists of English comments is obtained from Kaggle which contains about 7733 comments, example of English comments is shown in Table 2.

**TABLE 2:** Example of moderate, not depressed, and severe in English.

Comments	Labels
Age no job sleeping thinking of suicide	Severe
Logging out I need to study	Not depressed
I forgot how to sleep	Moderate

**FIGURE 2:**
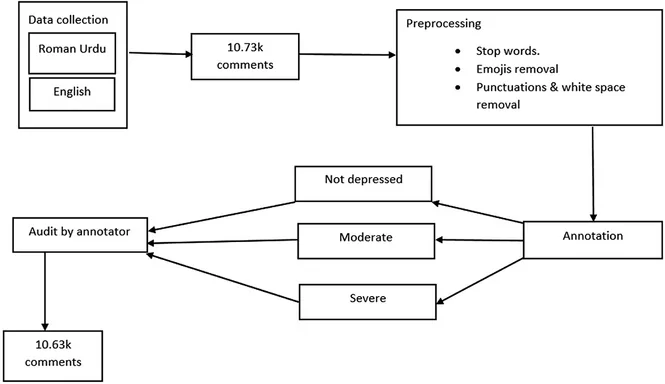
The process of data collection and annotation.

We merged these two datasets and turned them into one corpus to achieve our research objectives. Figure 2 shows the process of data collection and annotation.

### 3.2 Data preprocessing

The steps of preprocessing are explained in detail below.

#### 3.2.1 Lower case conversion

This is an initial and very simple approach in data preprocessing. We have converted our corpus to lower case, which is a very important step to maintain consistency and get the best results.

#### 3.2.2 Removal of emojis

Although emojis are considered very important for sentiments and emotions, but in this research, we focus on the prediction of depression through text only. Due to this all emojis have been deleted.

#### 3.2.3 Punctuations, stop words and white spaces

We removed punctuation, stop words and white spaces from the corpus to make it more understandable.

### 3.3 Annotation

Data annotation is sometimes called data labelling. In this process, data is labelled with different tags or classes. This step is very castigatory because it has a direct impact on accuracy. Annotation of data can be done automatically or manually by humans. The manual annotation process is expensive, due to this automatic tools and techniques are used for annotation. But in automatic annotation, we face some obstructions in which low accuracy is at the top. To avoid these obstructions, we adopt manual annotation.

We have manually annotated each comment in the dataset and classified it into three labels: moderate, not depressed, and severe.

### 3.4 Labels encoding process

In this process, labels are converted into numeric form to make them easy to understand for machine learning models.

We have converted the labels (not depressed, moderate and severe) from text to numbers (0, 1, 2). Make it readable for machine learning algorithms. We use LabelEncoder () to convert our labels from text to numbers.

### 3.5 Feature extraction

Feature extraction is used to convert the raw data into the desired form of data for modelling purposes and make it easy to understand for machine learning models. Feature extraction is used to reduce noise, remove irrelevant and redundant data from dataset and to extract useful features to train machine learning algorithms.

To train our models and to get the best result and accuracy, we create a new set of features‐by using the count‐vectorization method of feature extraction.

- Count‐vectorization

Count vectorization is an amazing tool of the scikit‐learn library that reads data and converts text into vectors (numbers) based on the frequency of words. Each word in the text is divided into separate columns and their vectors are assigned to them. We have extracted features by using count‐vectorization, that convert high dimensional dataset to lower dimensional dataset to make it readable for machine learning algorithms.

### 3.6 Training and testing

The training and testing process is the most essential step. It affects the achievements of machine learning models. In this phase, data is split into two parts: training and testing. The training data is bigger than the testing data. We split our dataset by 20–80%. It means that we use 80% of the data to train the models and the remaining 20% is used for testing.

### 3.7 Classification

Four famous machine learning algorithms were investigated on our created dataset.

#### 3.7.1 Support vector machine (SVM)

Support vector machine is a supervised machine learning classifier. It is usually used for classification and regression analysis. Kernels are variations of SVM that are used to modify the data to find an optimal result. A linear kernel is used in this study for classification.

#### 3.7.2 SVM RBF

The radial basis function is frequently used in SVM. When the data points are large number RBF is used to generate a flat surface.

#### 3.7.3 Random Forest (RF)

Random Forest is an ensemble learning model that is used for classification and regression. Random Forest combines the outcomes of multiple decision trees. And after combining the outcomes, it generates a single result.

#### 3.7.4 BERT

Bidirectional encoder representations from transformers stand as an open‐source machine learning framework design for the field of natural language processing (NLP). In NLP, BERT is used for classification tasks like sentiment analysis. The main aim of BERT is to classify the text into different categories.

### 3.8 Performance evaluation

Numerous metrics are used to calculate the model's performance. We use some performance metrics to estimate the performance of our models.

Confusion metric is visualized in the form of table. Confusion metric was put in, to represent the achievements of our prediction models. It authorizes us to measure its essential factors, accuracy, precision, recall and F1 score by using its values, TP, TN, FN, and FP.

TP = True positive.
TN = True negative.
FN = False negative.
FP = False positive.

Accuracy is the most frequently used metric.

(1) $$\mathrm{Accuracy}\;=\frac{\mathrm{TP}\;+\;\mathrm{TN}}{\mathrm{TP}\;+\;\mathrm{FN}\;+\;\mathrm{TN}\;+\;\mathrm{FP}}\;$$

Precision is interpreted as the proportion of true positives to comments.

(2) $$\mathrm{Precision}\;=\frac{\mathrm{TP}}{\mathrm{TP}\;+\;\mathrm{FP}}\;$$

Recall is interpreted as the proportion of TP to the verified positive net result.

(3) $$\mathrm{Recall}\;=\frac{\mathrm{TP}}{\mathrm{TP}\;+\;\mathrm{FN}}\;$$

By adopting the harmonic mean of precision and recall, F1 score merges them into one metric.

(4) $$F1-\mathrm{score}\;=\;2\times \frac{\mathrm{percision}\;\times \;\mathrm{recall}}{\mathrm{percision}\;+\;\mathrm{recall}\;}$$

## 4 RESULTS

This section includes the results of our investigated models with respect to predicting depression.

### 4.1 SVM

We investigate different algorithms to check the performance of our dataset. SVM achieve the best result with the accuracy of 0.84% on our created dataset.

**FIGURE 3:**
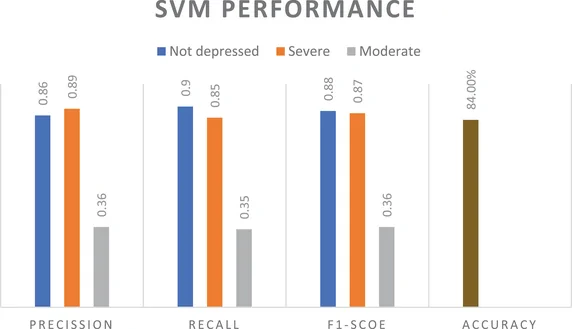
Performance of SVM.

Our dataset contains three classes in which not depressed class achieve 0.86% of precision, 0.90% of recall and 0.88% of F1 score, severe class achieve the precision of 0.89%, recall of 0.85%, and F1 score of 0.87% and the moderate class achieve 0.36% of precision, 0.35% of recall and 0.36% of F1 score. Result is shown in Figure 3.

### 4.2 SVM RBF

The result of Support Vector Machine Radial Basis Function is slightly less than the Support Vector Machine. SVM RBF achieves an accuracy of 82%. 2% less than the Support Vector Machine.

**FIGURE 4:**
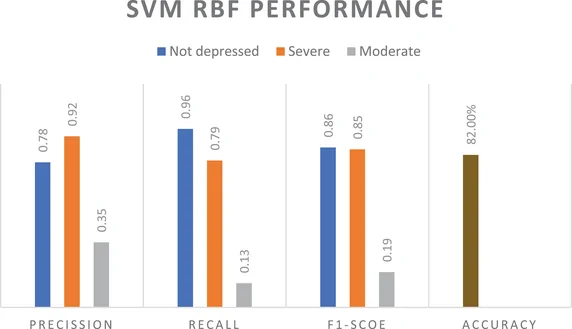
Performance of SVM RBF.

We noticed that moderate and severe class achieve 6%, 4% higher precision than Random Forest. The result is shown in Figure 4.

### 4.3 RF

**FIGURE 5:**
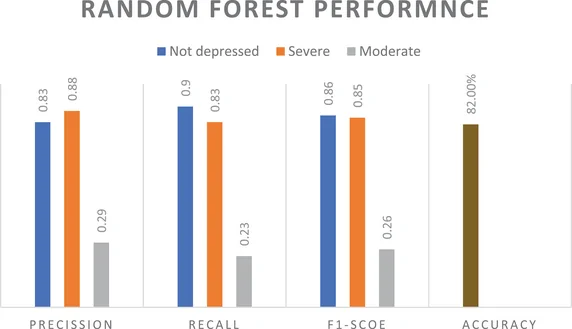
Performance of Random Forest.

The points of dataset are 2132. Random Forest achieves the accuracy of 82%, and the not depressed class achieves 5% higher precision than SVM RBF as shown in Figure 5.

### 4.4 BERT

**FIGURE 6:**
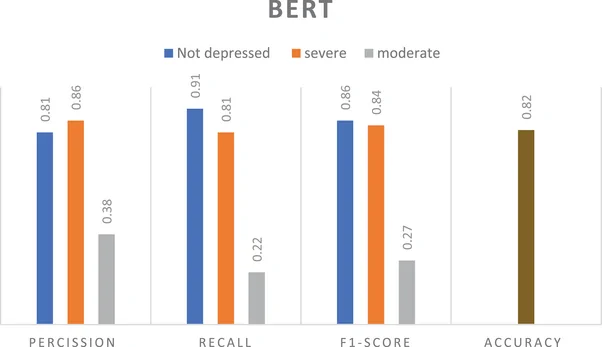
Performance of BERT.

BERT achieves the accuracy rate of 0.82% and not depressed class achieved only 1% higher recall than Random Forest as shown in Figure 6.

## 5 DISCUSSION

This section includes the results of our investigated models in respect of predicting depression. Table 3 shows the obtained results which includes accuracy, precision, recall and F1 score. To predict the depression through structural and non‐structural languages by using different machine learning models SVM achieve the best result with the accuracy of 0.84%, in which not depressed class achieve 0.86% of precision, 0.90% of recall and 0.88% of F1 score, severe class achieve the precision of 0.89%, recall of 0.85%, and F1 score of 0.87% and the moderate class achieve 0.36% of precision, 0.35% of recall and 0.36% of F1 score. Results are shown in Table 3.

**TABLE 3:** Performance evaluation.

			Metrics			
No.	Classifiers	Labels	Precision	Recall	F1‐Score	Accuracy
		Not depressed	0.86	0.90	0.88	
1	SVM	Moderate	0.36	0.35	0.36	0.84%
		Severe	0.89	0.85	0.87	
		Not depressed	0.78	0.96	0.86	
2	SVM RBF	Moderate	0.35	0.13	0.19	0.82%
		Severe	0.92	0.79	0.85	
		Not depressed	0.83	0.90	0.86	
3	Random Forest	Moderate	0.29	0.23	0.26	0.82%
		Severe	0.88	0.83	0.85	
		Not depressed	0.81	0.91	0.86	
4	BERT	Moderate	0.38	0.22	0.27	0.82%
		Severe	0.86	0.81	0.84	

SVM RBF achieves the result with an accuracy of 0.82%, in which the not depressed class achieves 0.78% precision, 0.96% recall and 0.86% F1 score, severe class achieves precision of 0.92%, recall of 0.79%, and F1 score of 0.85% and the moderate class achieves 0.35% precision, 0.13% recall and 0.19% F1 score.

And Random Forests achieve the result with an accuracy of 0.82%, in which the not depressed class achieves 0.83% precision, 0.90% recall and 0.86% F1 score, severe class achieves the precision of 0.88%, recall of 0.83%, and F1 score of 0.85% and the moderate class achieves 0.29% precision, 0.23% recall and 0.26% F1 score.

BERT achieves the result with an accuracy of 0.82%, in which the not depressed class achieves 0.81% precision, 0.91% recall and 0.86% F1 score, severe class achieves the precision of 0.86%, recall of 0.81%, and F1 score of 0.84% and the moderate class achieves 0.38% precision, 0.22% recall and 0.27% F1 score.

## 6 CONCLUSION

In south Asian regions, especially in Pakistan and India, Roman Urdu is very famous among the people through which they can communicate with each other on digital media platforms such as Twitter, Facebook, Instagram, and SMS.

But by analysing the previous studies we were unable to find any significant contribution in predicting the depression through Roman Urdu. To the best of our knowledge we were unable to find any dataset to predict depression in Roman Urdu, or Roman Urdu along with English.

In this research, first we manually created a dataset of Roman Urdu for non‐structural language, which consists of 3k comments, and for structural language, an English language dataset was obtained from Kaggle. We merged these two datasets and turned them into one corpus to achieve our research objectives. Features are extracted by using count vectorization. Then the data is divided into 2 parts: training and testing, 80% of the data have been used for training and the remaining 20% have been used for testing purpose. In the final phase selected models (SVM, SVM RBF, RF, and BERT) are investigated, and we identify various ML algorithms, accuracy, precision, recall, and F‐measure. Out of these models SVM achieves the best result with an accuracy of 0.84% on our created dataset.

As can be seen in the results, the result of the moderate class is less than that of the other classes. The reason for this is the lack of data for this class. In future work, the authors will employ advanced hybrid machine learning models like [27, 28, 29, 30, 31] to improve the accuracy in depression prediction in European countries.

## AUTHOR CONTRIBUTIONS

**Filza Rehmani**: Conceptualization; formal analysis; methodology; writing—original draft. **Qaisar Shaheen**: Investigation; project administration; supervision. **Muhammad Anwar**: Investigation; methodology; validation; writing—review and editing. **Muhammad Faheem**: Data curation; validation; visualization; writing—review and editing. **Shahzad Sarwar Bhatti**: Investigation; resources; validation.

## CONFLICT OF INTEREST STATEMENT

The authors declare no conflicts of interest.

## Data Availability

The dataset used in this study is available upon request from the author Filza Rehmani at filzarehmani@gmail.com. Due to privacy and ethical considerations, access to the dataset is subject to approval by the institutional review board (IRB) and compliance with relevant data protection regulations.

## References

[1] Depression . Accessed 15 Mar 2023. https://www.who.int/news‐room/fact‐sheets/detail/depression

[2] Are suicide rates higher for people with bipolar disorder? Healthline. Accessed Mar 16, 2023 https://www.healthline.com/health/suicide‐and‐bipolar

[3] Biggest social media platforms 2023 | Statista. Accessed Mar 16, 2023. https://www.statista.com/statistics/272014/global‐social‐networks‐ranked‐by‐number‐of‐users/

[4] Bilal, A. , Rextin, A. , Kakakhel, A. , Nasim, M. : Roman‐txt: Forms and functions of roman Urdu texting. In: Proceedings of the 19th International Conference on Human‐Computer Interaction with Mobile Devices and Services , pp. 1–9. Association for Computing Machinery, New York, NY (2017). 10.1145/3098279.3098552

[5] Rafae, A. , Qayyum, A. , Moeenuddin, M. , Karim, A. , Sajjad, H. , Kamiran, F. : An unsupervised method for discovering lexical variations in roman Urdu informal text. In: Proceedings of the 2015 Conference on Empirical Methods in Natural Language Processing , pp. 823–828. Association for Computational Linguistics, USA (2015). 10.18653/v1/D15-1097

[6] Gallup . Accessed: 15 Mar, 2023. https://gallup.com.pk/bb_old_site/Polls/7‐12‐09.pdf

[7] Aldarwish, M.M. , Ahmad, H.F. : Predicting depression levels using social media posts. In: Proceedings of the 2017 IEEE 13th International Symposium on Autonomous Decentralized System (ISADS) , pp. 277–280. IEEE, Piscataway, NJ (2017). 10.1109/ISADS.2017.41

[8] Siddiqui, S. , Singh, T. : Social media its impact with positive and negative aspects. IJCATR 5(2), 71–75 (2016). 10.7753/IJCATR0502.1006

[9] Chiong, R. , Budhi, G.S. , Dhakal, S. , Chiong, F. : A textual‐based featuring approach for depression detection using machine learning classifiers and social media texts. Comput. Biol. Med. 135, 104499 (2021). 10.1016/j.compbiomed.2021.104499 34174760

[10] Depression . Accessed: 15 Mar 2023. https://www.who.int/news‐room/fact‐sheets/detail/depression

[11] Islam, M.R. , Kabir, M.A. , Ahmed, A. , Kamal, A.R.M. , Wang, H. , Ulhaq, A. : Depression detection from social network data using machine learning techniques. Health Inf. Sci. Syst. 6, 1–12 (2018) 30186594 10.1007/s13755-018-0046-0 PMC6111060

[12] AlSagri, H.S. , Ykhlef, M. : Machine learning‐based approach for depression detection in twitter using content and activity features. IEICE Trans. Inf. Syst . E103.D(8), 1825–1832 (2020)

[13] Wang, X. , et al.: Depression risk prediction for Chinese microblogs via deep‐learning methods: Content analysis. JMIR Med. Inf. 8(7), e17958 (2020). 10.2196/17958 PMC7424493 32723719

[14] Uddin, M.Z. , Dysthe, K.K. , Følstad, A. , Brandtzaeg, P.B. : Deep learning for prediction of depressive symptoms in a large textual dataset. Neural Comput. Appl. 34(1), 721–744 (2022). 10.1007/s00521-021-06426-4

[15] Yang, T. , et al.: Fine‐grained depression analysis based on Chinese micro‐blog reviews. Inf. Process. Manage. 58(6), 102681 (2021). 10.1016/j.ipm.2021.102681

[16] Ullah, K.A. , Rehman, F. , Anwar, M. , Faheem, M. , Riaz, N. : Machine learning‐based prediction of osteoporosis in postmenopausal women with clinical examined features: A quantitative clinical study. Health Sci. Rep. 6, e1656 (2023) 37900094 10.1002/hsr2.1656 PMC10600334

[17] Dharma, E.M. , Heryadi, Y. , Lukas, W.S. , Wibowo, A. : Predicting depression levels using back propagation neural network. J. Comput. Sci. 18(3), 151–161 (2022) 10.3844/jcssp.2022.151.161

[18] Vázquez‐Romero, A. , Gallardo‐Antolín, A. : Automatic detection of depression in speech using ensemble convolutional neural networks. Entropy 22(6), 688 (2020). 10.3390/e22060688 33286460 PMC7517226

[19] Naseem, S. , Alhudhaif, A. , Anwar, M. , Qureshi, K.N. , Jeon, G. : Artificial general intelligence based rational behavior detection using cognitive correlates for tracking online harms. Pers. Ubiquitous Comput. 27(1), 119–137 (2023)

[20] Sturim, D. , Torres‐Carrasquillo, P.A. , Quatieri, T.F. , Malyska, N. , McCree, A. : Automatic detection of depression in speech using Gaussian mixture modeling with factor analysis. In: 2011 INTERSPEECH, pp. 2981–2984. Massachusetts Institute of Technology, Lexington, MA (2011)

[21] Bilal, M. , Ali, G. , Iqbal, M.W. , Anwar, M. , Malik, M.S.A. , et al.: Auto‐prep: Efficient and robust automated data preprocessing pipeline. IEEE Access 10, 107764–107784 (2022)

[22] Cohn, J.F. , et al.: Detecting depression from facial actions and vocal prosody. In: *Proceedings of the* 2009 3rd International Conference on Affective Computing and Intelligent Interaction and Workshops , pp. 1–7. IEEE, Piscataway, NJ (2009). 10.1109/ACII.2009.5349358 PMC3296481 21278824

[23] Cha, J. , Kim, S. , Park, E. : A lexicon‐based approach to examine depression detection in social media: The case of Twitter and university community. Humanit. Soc. Sci. Commun. 9(1), 325 (2022). 10.1057/s41599-022-01313-2 36159708 PMC9491270

[24] Ochnik, D. , et al.: A comparison of depression and anxiety among university students in nine countries during the COVID‐19 pandemic. JCM 10(13), 2882 (2019), (2021). 10.3390/jcm10132882 PMC8269122 34209619

[25] Urdu language .: Encyclopaedia Britannica. https://www.britannica.com/topic/Urdu‐language. Accessed

[26] Ali, G. , Anwar, M. , Nauman, M. , Faheem, M. , Rashid, J. : Lyme rashes disease classification using deep feature fusion technique. Skin Res. Technol. 29, e13519 (2023) 38009027 10.1111/srt.13519 PMC10628356

[27] Alarood, A. A. , et al.: Secure medical image transmission using deep neural network in e‐health applications. Healthc. Technol. Lett. 10(4), 87–98 (2023) 37529409 10.1049/htl2.12049 PMC10388229

[28] Ali, G. , et al.: A hybrid convolutional neural network model for automatic diabetic retinopathy classification from fundus images. IEEE J. Transl. Eng. Health Med. (2023)

[29] Faheem, M. , Al-Khasawneh, M. A. Multilayer cyberattacks identification and classification using machine learning in internet of blockchain (IoBC)‐based energy networks. Data in Brief. 110461 (2024) 38774244 10.1016/j.dib.2024.110461 PMC11106829

[30] Kawoosa, A. I. , et al.: Using machine learning ensemble method for detection of energy theft in smart meters. IET Gener. Transm. Distrib. 17(21), 4794–4809 (2023)

[31] Ullah, K. A. , et al.: Machine learning‐based prediction of osteoporosis in postmenopausal women with clinical examined features: A quantitative clinical study. Health Sci. Rep. 6(10), e1656 (2023) 37900094 10.1002/hsr2.1656 PMC10600334

